# A Case of Obstetrics

**Published:** 1879-04

**Authors:** E. A. Cobleigh

**Affiliations:** Athens, Tennessee


ATLANTA
Medical and Surgical Journal.

VOL. XVII.] APRIL—1879. [No. 1

### Original Communications.

### A CASE OF OBSTETRICS.

BY E. A. COBLEIGH, M.D., ATHENS, TENNESSEE.

Every medical practitioner meets cases of disease presenting peculiar features, and often finds himself confronted by new and inexplicable morbid manifestations. So, also, the physician not unfrequently gets caught in some unexpected emergency, which calls on him to put forth his best efforts to save life or relieve suffering. Often he finds old and tried methods of procedure unavailing for the accomplishment of these desirable ends, and resorts to unprecedented means for bringing about his object, sometimes succeeding happily; again, chagrined by defeats, miserably failing. Mutual exchange of experience has largely made our profession what it is—no one individual's labor or experience being worth much when disconnected from his personal imbibations of knowledge from outside sources—and it is the duty of each one of us to communicate anything new or novel which has proved available for good in our hands.

Wherefore, though a like proceeding may not be necessary in the hands of one physician out of every thousand, I deem the following case and its treatment sufficiently novel to merit a report to my brethren of the profession generally. In a similar case, and with the same surroundings, a man would give anything to be able to call to mind some readily accessible and tested remedy for the relief of a condition which is tending rapidly and surely to death, and which has resisted all approved means of cure within reach of the anxious attendant. I reported the case at the last meeting of our medical society, though it occurred to me some time ago—it having been brought feebly to mind by a more recent parturition, where I was also forced to resort to unusual and rather severe measures, though not to so *dernier* a resort as the one I will now lay before you.

Was called at eight o'clock, P.M., on Nov. 28th, 187–, to attend Mrs. F. B. in her second pregnancy at term. Mrs. B. was a lady of fine build and appearance, medium height, about thirty years of age, and of good constitution. She was neither plethoric, anæmic, nor nervous, and in both of her pregnancies had been free from unpleasant symptoms, so common in this condition. She had been almost exempt from nausea, had had no marked neuralgic disturbances, no weakness, and very little evidence of any inconvenience at all. Her first child was at this time about three years old. She had had no menstrual trouble since puberty, and no miscarriage. Married nearly five years.

This was my first attendance on her, and the above facts were ascertained after reaching her residence, some five miles in the country. I found her in bed, everything prepared nicely for the occasion, and my patient calm and cheerful. Pains had commenced regularly about eight hours before sending for me, gradually increasing and recurring at shorter intervals. They were now coming on about every eight or ten minutes. There was no nausea or nervous perturbation. On digital examination, found a large, well-formed pelvis, the soft tissues in fine condition, and a decided right obliquity of the uterus. This I rectified by placing the lady on her left side. The cervix was fully dilated, liquor-amnii in small quantities and unevacuated, cephalic presentation in the first position of Baudelocque, and everything thus far presaging a speedy and safe delivery.

On leaving home I had hastily thrust into my pocket my ergot vial and a bottle of chloroform, together with my case of pocket instruments. These were all I had with me to use in an emergency during the labor, should any such occur, which I did not apprehend.

The parturient process progressed steadily but rather slowly, so I ruptured the membranes. From this moment the labor was rapid. In a few minutes the head was born, followed, almost without any cessation of pain, by the shoulders and body of a large and hearty male child, which weighed nine pounds and a fraction. It is customary with me, when there is no contra-indication to its use, to administer ergot in every case of labor so soon as the occiput presses on the perineum; but matters progressed so rapidly here that I had no opportunity, despite my efforts to retard delivery, to attend to giving the usual dose.

The uterus contracted down on to the placenta nicely, and as the infant was crying vociferously, I proceeded to divide the cord. Having passed the child to an attendant in waiting therefor, I had a fluid drachm of Tilden's F. E. ergot administered to my patient, and, to my surprise, found it to be the entire contents of the bottle. With scarcely a delay of one minute I re-examined Mrs. B., to find the placental mass already occupying the vagina, and at once extracted it entire, with the membranes. Following the after-birth, came an enormous mass of clots and a stream of uncoagulated blood, as if from an open flood-gate. Passing my hand forthwith to the uterine cavity, I attempted, by conjoined manipulation, to coax contraction. The whole organ had relaxed seemingly to its previous gravid size. Partial contraction occurred in a moment, but not enough to control measureably the flooding. And then a series of alternative and irregular contractions and expansions—a sort of vibration of the uterine walls—took place between my fingers, profuse hemorrhage continuing all of the time. I now tried, severally, pressure, internal titillations, external frictions, aortic compression, cold cloths to vulva and abdomen and spine, and, lastly, not only was the infant applied to the breast, but I freely used cold douches and even passed a cold wet cloth to the uterine fundus.

There was nothing which could answer as a hæmostatic agent (not even alum) in the house—no ice to be had, though the weather was cold, no syringe obtainablė, no time to send for remedies or counsel. It was one of those cases where the physician can't consult text books—can't even get the desired remedies to use, but must rely on his own unaided judgment, do his best with the limited means at his command, keep cool and assume of necessity the weighty responsibility involving life or death to one who has confided her safety to his keeping. I felt the immense interest at stake, and saw my patient evidently nearing eternity, despite every effort I was able to put forth in her behalf. Never had I witnessed or heard of a case exactly parallel to the one in hand.

Mrs. B. fainted, but speedily revived. The womb had now ceased its wavy action and assumed a tonic state of semi-contraction, yet hemorrhage continued, though not so profuse. Her face was blanched, she complained of blindness—partial, not comp'ete—tinnitus aurium, and other symptoms of profound systemic exsanguination. There were no spirits or other stimulants to be had, but a messenger had already been dispatched to a neighbor's house for brandy. The pillows were taken from beneath her head, the foot of the bed was raised, and hot flannels applied to the body.

Just at this crisis I espied an iron poker standing near the fire-place. I had it heated in the blazing fire, which required but a few moment's time, and then, as a *dernier resort*, turned my patient on her side and cauterized the spinal region over a surface one-third the size of my hand. The eschar was, perhaps 4½ inches long by 1½ or 2 inches wide, and I was not particular to make it regular in outline, or calculate its size at the time. The effect was more than I had dared count on. Mrs. B. cried out in pain on applying the iron to her integument, but the uterus, almost immediately, contracted down and expelled my hand, the hemorrhage ceasing, of course. Nor was the effect merely momentary, for the contraction continued firm until I left the house, two hours later.

But the effect of the cautery was even more than oxytoxic in character. The pain proved a powerful stimulant and kept up the failing circulation of my patient until some spirits were obtained and administered.

It is unnecessary to particularize regarding the after treatment in this case. Stimulants, nourishment and tonics accomplished the work of recovery, and Mrs. B. lives to-day to attest the wonderful powers of actual cautery in arresting post-partem hemorrhage. The burned surface was slow in healing, but not more so than any other abraded surface would have been in so debilitated and ex-sanguine a body as hers, and though her own recovery was slow, it was perfect. She has since been delivered at term without any puerperal complication whatever.

I report this case more as a novelty than otherwise, for it is not often that the accoucheur is so situated as to necessitate resort to such harsh measures as actual cautery. Yet, uterine inertia does frequently occur, and perhaps some professional brother may be so placed as to find remedies unattainable, and the hot iron a ready way to save his patient and help himself out of a trying position. Yet, it is possible that even this *dernier resort* may not uniformly have the effect it did in my hands. My experience with it is limited to this one case, and I hope that if it must be further tested, the necessity may fall on other shoulders than mine.

